# Surpassing the advanced comes from continuous accumulation

**DOI:** 10.1007/s13238-014-0056-x

**Published:** 2014-05-05

**Authors:** Baoyuan Zhang, Jing Qu, Nur Estya Binte Rahman

**Affiliations:** 1 Beijing Institutes of Life Science, Chinese Academy of Sciences, Beijing, 100101 China; 2 Temasek Life Sciences Laboratory, 1 Research Link, National University of Singapore, Singapore, 117604 Singapore

As a famous Chinese saying goes, “A journey of a thousand miles begins with a single step”. It teaches us that perseverance and steadfastness are absolutely essential for success and progress. This philosophy has been illustrated by an exemplary Chinese microbiologist, Taosheng Chen.

Taosheng Chen was one of the pioneers in modern industrial microbiology research in China. His pioneer work in improving the traditional Chinese brewing techniques and the establishment of modern industrial microbiology technology has been regarded as an outstanding contribution in the development of Chinese industry. Specifically in alcohol production technology, he had successfully surpassed international experts twice, marking them as commendable events in the history of Chinese alcohol fermentation.



The beginnings of Taosheng Chen’s breakthrough began during the Chinese economic depression in the early 1920s. Shandong Pu Yi Winery was established in 1922, with the primary aim to revitalize the national industry. Shandong Pu Yi Winery was the first alcohol plant established by the Chinese. In its rudimentary stages, production equipment were ordered from Japan, which includes alcohol distillation towers, cooking pots, pots for glycosylation, coolers and other components. Therein, 30 cement fermentation pools (painted with asphalt) were built specifically for alcohol production. Notably, the plant owner hired a Japanese expert, Watanabe, for the operation of the alcohol production technology. At that time, Taosheng Chen, only 24 years old, was a technical staff of the plant. He examined the molasses fermentation stored in the pits and found that fermented mash produced no bubble, without the scent of alcohol. However, this result did not hinder him. By adding sulfuric acid into molasses, he smelled the stench of decomposing molasses after pressure cooking. After removing the stench, he diluted the molasses with water, added yeast for fermentation, and eventually achieved the desired fermented products—alcohol. Through thorough analysis, he found that the molasses was easily infected by butyric acid bacteria during storage. The bacteria decomposed glucose into butyric acid and hydrogen in the metabolic processes. The hydrogen reduced nitrate in the molasses, resulting it to release nitric oxide which is an inhibitor of alcoholic fermentation. This important discovery elucidated the reasons the alcoholic fermentation ceased and was incomplete to produce the desired alcohol products. Taosheng Chen was quite delighted with this finding and gained more confidence in his fermentation technology methods. Although Japanese expert Watanabe was well-versed with cereal fermentation technology, he was not familiar with using beet molasses to ferment alcohol. The plant owners trusted Watanabe but did not recognize Taosheng Chen’s potential in fermentation technology. As Watanabe experienced continuous failures in the beet molasses fermentation technology, Taosheng Chen decided to contribute his ideas confidently in refining the fermentation technology of beet molasses. He illustrated that the molasses could be pre-treated and fermented through his own methodology. Results indicated that one hundred pounds of molasses could produce 24 pounds of alcohol (with a fermentation efficiency as high as 85 %). After the successful commissioning of the production, the plant held a special celebration meeting and announced that Taosheng Chen was promoted to be an engineer and technical director of the plant, rewarded with higher remuneration. Furthermore, the company’s technical adviser German expert Lindemann and Japanese expert Hori Soichi also congratulated him for the internationally advanced achievement, and jointly signed the certification letter.

Another breakthrough made by Taosheng Chen that surpassed international experts was accomplished in 1933. Several Chinese patriots abroad decided to set up a company in Shanghai where they imported equipment abroad to produce alcohol using sugarcane molasses from Nanyang, as its raw material. The expected daily alcohol production was 20,000 gallons (equivalent to an estimated 90,922 liters), and with that efficiency of alcohol production, the company aimed to be the first and leading alcohol production company in the Far East. In the initial stages of alcohol production, the plant hired a British engineer, Brown, to manage the technical work. However, Brown did the fermentation without a small scale trial. Several months passed, the production was still unable to achieve the desirable alcohol yield. After which, Taosheng Chen went to the factory, analyzed the component of sugarcane molasses with other technical staffs, and conducted small scale fermentation trials. They found that the sugarcane molasses lacked nitrogen sources. Sufficient nitrogen supplement was needed in the fermentation. Otherwise, yeast fermentation could not produce any alcohol. With this in mind, they repeated fermentation experiments many times in order to select a perfect yeast strain with high capacity in sugarcane molasses fermentation. With the results of these small scale trials, the first big scale fermentation thereafter proved to be successful and the daily production improved and doubled. When alcohol rolled out from the distillation machine pipe, the general manager of the company said, “The Chinese people are really great!”

Research work often encounters a paradoxical problem. Without long-term and persistent theoretical accumulation and practical exploration, people will not find the paths of truth. Once, someone ever asked Taosheng Chen, “You have studied modern industrial microbiology for decades and accomplished numerous achievements. How did you do it?” He replied, “To someone who is engaged in scientific research, there are four prerequisites: first, he should have a solid basic knowledge; second, he must be good at summing up experiences of predecessors; third, he should have research plans and summarize the learning step by step; fourth, whatever the difficulties are, he must not give up. Persistent efforts lead to success”. These remarks are still significant for today’s research work. Nowadays, most of the emerging industries are high-tech ones, while traditional industries based on fundamental research often lag behind. They require more innovation to overcome difficulties. Taosheng Chen also pointed out, “If anyone can thoroughly study microbes in wine, he can be awarded the Nobel Prize.” It also demonstrates that we must not forget and pay more attention on basic research. Only by continuous persistence and reflection can we realize self-transcendence and accomplish achievements in the future.
